# Multilevel *omic* data integration in cancer cell lines: advanced annotation and emergent properties

**DOI:** 10.1186/1752-0509-7-14

**Published:** 2013-02-19

**Authors:** Yuanhua Liu, Valentina Devescovi, Suning Chen, Christine Nardini

**Affiliations:** 1 Key Laboratory of Computational Biology, CAS-MPG Partner Institute for Computational Biology, Shanghai Institutes for Biological Sciences, Chinese Academy of Sciences, Shanghai, China; 2 First Affiliated Hospital of Suzhou University, Jiangsu Institute of Hematology, , Suzhou, China

**Keywords:** Multi-omic, Emergent property, Factor analysis, Linear discriminant analysis, NCI-60 cell panel

## Abstract

**Background:**

High-throughput (omic) data have become more widespread in both quantity and frequency of use, thanks to technological advances, lower costs and higher precision. Consequently, computational scientists are confronted by two parallel challenges: on one side, the design of efficient methods to interpret each of these data in their own right (gene expression signatures, protein markers, etc.) and, on the other side, realization of a novel, pressing request from the biological field to design methodologies that allow for these data to be interpreted as a whole, i.e. not only as the union of relevant molecules in each of these layers, but as a complex molecular signature containing proteins, mRNAs and miRNAs, all of which must be directly associated in the results of analyses that are able to capture inter-layers connections and complexity.

**Results:**

We address the latter of these two challenges by testing an integrated approach on a known cancer benchmark: the NCI-60 cell panel. Here, high-throughput screens for mRNA, miRNA and proteins are jointly analyzed using factor analysis, combined with linear discriminant analysis, to identify the molecular characteristics of cancer. Comparisons with separate (non-joint) analyses show that the proposed integrated approach can uncover deeper and more precise biological information. In particular, the integrated approach gives a more complete picture of the set of miRNAs identified and the Wnt pathway, which represents an important surrogate marker of melanoma progression. We further test the approach on a more challenging patient-dataset, for which we are able to identify clinically relevant markers.

**Conclusions:**

The integration of multiple layers of omics can bring more information than analysis of single layers alone. Using and expanding the proposed integrated framework to integrate omic data from other molecular levels will allow researchers to uncover further systemic information. The application of this approach to a clinically challenging dataset shows its promising potential.

## Background

Due to the rapid advances in high-throughput technologies, the quantitative monitoring of various biological molecules at the genomic scale (transcriptomics, post-transcriptomics and proteomics, i.e. *omics*) is now easily made available to number of laboratories at quickly dropping costs. However, any single *omic* screen cannot fully unravel the complexity of a biological entity: integration of multiple layers of information, (multi-*omic*) is therefore required to understand more of these systems.

**Figure 1:**
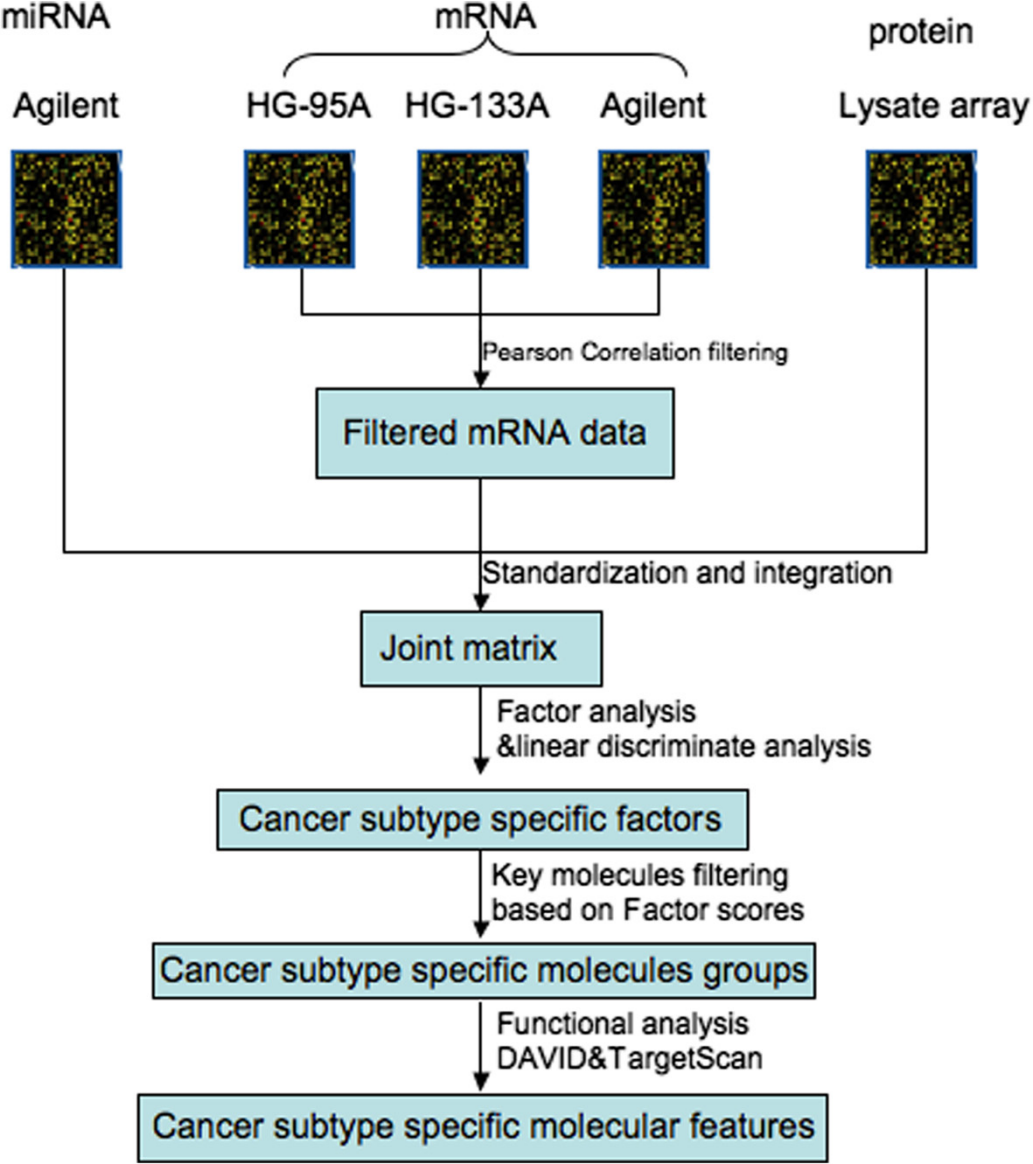
**Flowchart of data integration analysis.** General outline of data integration, where three steps are involved: (1) mRNA, miRNA and protein omic data are standardized and merged into one matrix (joint matrix); (2) FA is done on the joint matrix to identify the tissue-specific factors, through which key molecules, including mRNAs, miRNAs and proteins, are filtered; (3) Functional analysis is performed using DAVID to extract cancer-related features.

This study presents first the integrated analysis (transcriptional, post-transcriptional and translational data, [1,2]) of the multi-panel cancer dataset NCI-60, a set of 60 diverse human cancer cell lines derived from 9 different tissues (http://discover.nci.nih.gov/cellminer/home.do). A scheme of the process is outlined in Figure 1. Building on our previous approach to integrate transcriptional and post-transcriptional data [3], the exemplar goal of this analysis is the identification of multimolecular features able to describe the tissues of origin of each sample. This dataset is also processed with non-joint approaches and alternative tools to quantify the information added by our proposed method. All these analyses and results are discussed in the body of the article. As a more challenging and practically relevant case we then tested our approach on the large multi-molecular ovarian cancer patients dataset (TGCA dataset, [4]). The results, based on our proposed approach, are presented in a separate Section at the end of this manuscript.

For data integration we used Factor Analysis (FA, [5]) applied directly on biological data without any *a priori* hypothesis. This is both the potential and the limitation of our approach: FA can isolate molecules that share patterns of co-variation, meaning that cross layers associations among molecules are already elaborated in the results proposed, as factors contain protein, miRNA and mRNA. However, this does not resolve the biological causes behind these associations: reasons of this common variance have then to be searched manually by an expert curator. Co-variation may therefore be attributed to the expression of genes under the same transcription factor, binding to the proper promoter sites spread across the genome, or to the repression of a function due to the silencing of co-expressed miRNAs, only to name a few. We made the conscious choice to leave interpretation to manual expert curation to allow maximum flexibility in the interpretation, spanning from annotations for functions or pathways to co-localization on the genome. Nevertheless, the use of *a priori* knowledge (namely the tumor tissue of origin for NCI-60 and clinical classifications for TGCA) to constrain via linear discriminant analysis (LDA, [6]) the relation between the latent variables under study and the factors obtained, eases the process of results’ interpretation, as it gives a phenotypic support to the molecular interpretation of the latent structures. We remark here that alternative approaches to constrain the factors model are possible and can lead to comparable results. In particular, LDA can be replaced with other classifiers such as Bayesian classifiers [7-9], Support Vector Machine [10], K-nearest-neighbor [11]. More details on alternative methods are discussed below and proposed in the Results and discussion section.

### Related work

The earliest attempts of data integration reported in literature analyze data from individual *omics* separately and only downstream of these parallel analyses results are merged (only to list a few [12-15]). This, although relevant, implies the loss of important properties which can only be uncovered when multiple *omic* dataset are viewed as a whole. The theoretical background behind this assumption lies in the definition of *emergent property* in Systems Theory, now becoming popular/familiar in Systems Biology [16-19]. Emergent properties indicate how some features of a system can only become observable when the system is studied as a whole and not as the sum of its parts. The justification -in a biological context- for the need to integrate mRNA, miRNA and protein expression has experimentally been quantified only recently [20].

More recent approaches have attempted to directly integrate multi-omic data. We cite here iCluster [21] as it is fundamentally based on the same principles as our own approach (FA). However, iCluster uses an unsupervised technique to identify the best factors (there named clusters) that is the minimization of the Proportion of Deviance (POD). Our approach, conversely, recommends LDA or other classifiers which aim at maximizing the accuracy of factors combinations in predicting some external categories (tissue of origin, response to therapy) it is therefore a supervised approach. Depending on the problem under study, supervised or unsupervised approaches may be necessary. We remark here that the ability to predict structures in the absence of external information (i.e. unsupervised) comes at higher computational costs for iCluster versus our approach (days versus minutes). PARADIGM [22] is another approach aiming at the integration of heterogeneous data and, additionally, at the inference of connections among the identified molecules. To date this method does not include miRNAs, and recovers connections on the bases of the signaling pathways it has been trained with (therefore excluding association due, for example, to co-localization on the genome). Integration of PARADIGM with our approach (provided they can both be input with the same data) could bring complementary information on multiomic analyses.

Finally, for the specific case of the NCI-60 cancer cell line dataset, since it has been deeply profiled for many types of research (drug response, chromosomal aberrations, mutational status, etc.) we highlight, among the wide range of literature existing, the following 3 researches, as they utilize as objective function for the evaluation of their results the appropriateness of the prediction of the tissue of origin. In [23] the authors perform miRNA profiling with the purpose of determining tissue-specific markers. We used these results as control of the coherence of our findings for miRNAs. Blower and co-workers [24] performed miRNAs screen on the NCI-60 cell lines, and suggest as future work to integrate various layers of omics to extract major information, therefore supporting the type of analysis here proposed. From their observations the authors conclude that cell line groupings based on miRNA expression are generally consistent with tissue type and with cell line clustering based on mRNA expression, although mRNA expression seems to be more informative. We will show in our work that indeed -when integrated- the two layers can bring even more information. Very recently, other authors [25] proposed a method to reconstruct association modules containing cancer aberrations drivers. The method evaluates a large number of variables including the effects of Copy Number Variations, genes mutations and methylations on the expression of mRNA and miRNA as well as the direct- and anti-correlation among mRNAs and between mRNAs and miRNAs. Although there are strong and well known limitations in the consideration of such direct types of correlations (see [3,24,26]) we think that the integration of our approach (for the mRNA, miRNA, protein association) with this one (for the DNA layer processing) could bring additional insight into the characterization of cancers, and can represent future work in this direction.

## Methods

### Materials

The NCI-60 is a set of 60 human cancer cell lines derived from 9 diverse tissues including melanomas (ME), leukemias (LE), breast (BR), renal kidney (RE), ovary (OV), nervous central system (CNS), non-small cell lung (LC), prostate (PR) and colon (CO) cancers (http://discover.nci.nih.gov/cellminer/home.do). Since 1992 these cell lines have been intensively studied and they have also, more recently, been processed with high-throughput technologies. The datasets here used are obtained from two different publications, where the same 60 NCI-60 cell lines are considered, prepared according to the same experimental protocol and sampled. Profiles of mRNA and miRNA can be found in [27] produced using Agilent technologies, while in [15] mRNA profiles are obtained with Affymetrix HG-U95A and HG-U133A chips and the protein level is analyzed by reverse-phase protein lysate arrays (RPLA).

### Data preprocessing

The three omic datasets (mRNAs, miRNAs and proteins) were downloaded from CellMiner (http://discover.nci.nih.gov/cellminer/home.do). The proprietary Affymetrix.CEL files were loaded and processed as described in the original publications, and finally mRNA and miRNA were treated with quantile normalization [28]. Since mRNA profiles are obtained from different labs and platforms, to account for unexpected variance or noise, we first filtered the mRNAs showing differential behavior between the 2 datasets (Pearson correlation >0.5). In general, multiple probes on a chips map on a single gene (Entrez Gene), therefore, for each gene, we calculated all the Pearson statistics between each probe in the two datasets [15,27]. For each gene, the maximum value was chosen as representative of the correlation of the two genes between the two studies, obtaining 27808 probes (16734 Entrez Gene IDs). In order to further compact and properly join the datasets, multiple probes treatment was then performed to cluster probes from the same gene (hierarchical clustering, cutoff height =0*.*6). For each cluster we choose the mean value to represent the expression of the gene, leading to a 24040×60 matrix from the above 27808 probes. We limited the number of mRNAs to probes that showed relatively high and diverse expression across the NCI-60, by calculating, for each mRNA probe (p) two values: maximum probe intensity, max(*p*), and probe inter-quartile range, *IQR*(*p*), across the dataset. In total, 6162 probes (out of 41,000) appeared in both the top half of max(*p*) and the top half of *IQR*(*p*). Multiple probes processing was also performed on the protein dataset and a 157×60 matrix (94 Entrez Gene IDs) was finally obtained. All the 365 miRNAs from [27] were used without any additional filtering. As a last step, the preprocessed datasets of mRNA, miRNA and protein were standardized across all samples using the mean as baseline: *x*=(*x*^*exp*^−*c*)/*c*, where *x*^*exp*^ represents the expression level and *c* the mean on all the samples of the same molecule. The three omic datasets were finally joined in a single (6162 + 157 + 365)×60 matrix on which FA was performed.

### Models definition

FA is a statistical method used to uncover the structure underlying a relatively large set of variables, which can be described as *X*=*FL* + *e*, where *X* is the omic joint matrix with samples representing the variables, *F* is the factors’ scores matrix representing the latent structure of *X*, *L* is the factors’ loadings matrix which shows the relationships between factors and variables, and *e* is the unique factors matrix. The maximum number(*n*) of meaningful latent features (factors) can be computed based on the general rationale that -upon factorization- the components of a matrix that explain less variance than the original standardized variables should be discarded, as they do not carry relevant information. Since the number obtained represents a maximum, after which factors may loose meaning and interpretability, it is useful to compute FA for all possible number of factors between 1 and *n*. Each of these FA results is named a model (M i, i=1,..n) here, and labeled with the corresponding number of factors (M1, Mn), each Model is therefore characterized by a growing number of factors named *F*_*j*_, *j*=1…*i*.

### Factors selection

Models were then selected based on their ability to distinguish cancers according to any of the relevant properties available (in our case tissue of origin for NCI-60, or response to thserapy for TGCA) using LDA. The *χ*^2^-test was used to estimate the significance of the LDA accuracy. The significant factors consist of lists of relevant molecules, weighted by their factors’ scores (threshold set to 2.6). The key point here is that these factors directly contain proteins, mRNAs and miRNAs that do not need further processing to be associated. These molecules’ groups are then annotated to ease the interpretation of the properties emerging from this joint analysis.

### Functional analysis

For each cancer subtype, the identified key mRNAs and proteins are annotated directly using DAVID, i.e. Gene Ontology (GO, [29]) PANTHER [30], BIOCARTA [31], KEGG [32] and RACTOME [33]. To examine the significance of the enrichment, a modified Fisher exact test (EASE score, [34]) was used to calculate the *p*-value, and FDR was further adopted to correct for multiple hypothesis testing (threshold 0.05), having the human genome as background. The miRNAs were annotated based on their targets identified via TargetScan [35].

### Comparison with other approaches

We compared our FA-based approach with other methods in two ways: i) joint analysis versus separate analysis and ii) FA-based joint approach versus other joint method.

In the first comparison, the separate analysis treated the mRNA, miRNA and protein datasets as separate matrices and imputed them separately in the pipeline FA+LDA, this outputs, for each omic layer a combination of tissue-specific factors. The key molecules in each omic layer are merged tissue-wise for functional annotation as described in Functional Analysis.

The second comparison tests the results on a different way of integrating the 3 omic layers using other classical methodologies, i.e. the combination of hierarchical clustering (HC) and SAM [36]. The joint (6162 + 157 + 320)×60matrix is used as input to HC via the function *hclust* in the R package *stats*, which results in different clusters (groups) specific to different tissues of origin. SAM is then used to group the clusters and to identify the key molecules (from 3 omic layers). Functional analysis is done similarly to the FA-based integrated method. Further description about the comparisons is discussed in Results and discussion section

## Results and discussion

For our dataset, the maximum number of meaningful factors is n=16. In the present manuscript we chose to focus on cancers tissue types, since they represent an easy-to-validate feature for which novel information can be rapidly integrated (see also Related work Section). LDA identifies as the best model to discriminate the samples with respect to the tissue of origin the 8-factor model (M 8) which can significantly discriminate the tissue of origins with accuracy (0.833). In particular, F1, F2, F3, F4, F5, F7 and F8 of this model can be used to discriminate respectively ME, CO, LE, RE, OV, LC and CNS cancers from other tissues of origin with significantly high accuracy (>0.9, see Table 1 for details). For the separate analysis, the best models to discriminate the tissue of origin are M 8 (among M 1-M 14) for mRNA (accuracy 0.783), M 10 (among M 1-M 20) for proteins (accuracy 0.833) and M 9 (among M 1-M 16) for miRNA (accuracy 0.633), details on the factors can also be found in Table 1.

**Table 1:** Models and factors that discriminate the tissue of origins via joint and separate analysis

**Methods**			**Joint**		**Separate**		
**Data**			**mRNA & Prot & miRNA**		**mRNA**	**Prot**	**miRNA**
**Best Model**			**M8**		**M8**	**M10**	**M9**
	ME		F1 (0.98, 3×10^−13^)		F1 (0.98, 3×10^−13^)	F4 (0.93, 7×10^−9^)	F1 (0.98, 3×10^−13^)
	CO		F2 (0.97, 8×10^−11^)		F2 (1, 7×9^−15^)	F8 (0.92, 1×10^−5^)	F2 (0.95, 2×10^−9^)
	LE		F3 (1.0, 9×10^−15^)		F3 (1, 9×10^−15^)	F1 (0.98, 1×10^−12^)	F3 (1.0, 9×10^−15^)
Tissue & Factor	RE		F4 (0.98, 7×10^−13^)		F4 (0.97, 7×10^−13^)	F10 (0.93, 1×10^−7^)	F7 (0.93, 3×10^−7^)
	OV		F5 (0.9, 2×10^−3^)		F5 (0.92, 1×10^−5^)	F3 (0.9, 3×10^−3^)	F9 (0.92, 8×10^−5^)
	LC		F7 (0.95, 8×10^−10^)		F7 (0.92, 8×10^−7^)	F2 (0.93, 3×10^−8^)	NA
	CNS		F8 (0.97, 5×10^−10^)		F8 (0.93, 2×10^−5^)	F7 (0.95, 5×10^−8^)	F8 (0.95, 1×10^−3^)

The first 3 rows in the table describe a hierarchy of information about the factor analyzed to extract information relevant for the tissue of origins listed in column 1. Namely: the type of analysis, that is joint or separate. Within the separate analysis 3 options are possible i.e. analysis of mRNA only, or miRNA only or proteins only. Finally, the factor analysis model (M) chosen to describe the tissue is noted in row 3, the number indicates the number of factors obtained from the analysis. Finally each cell in the matrix indicate which factor among the ones available in the model better describe each tissue type. In each cell, accuracy (acc.) and *p*-value related to the ability of the Factor to predict the tissue are reported.

In the rest of this section we report the biological meaning of the factor which loadings show the clearest relation with the tissue of origin in the integrated analysis, that is F 1 for melanomas (full molecules list in Additional file 1). In particular, we highlight how the method is able to identify two relevant types of information: a complete and up-to-date set of miRNAs -which involvement in tumorous processes is being increasingly appreciated-, and the crucial players in the Wnt pathway which importance in Melanomas is discussed in light of the most recent findings. Finally, a comparison with the results obtained with other approaches is also reported.

Before entering these details, we can generally observe an interesting *flow* of information, changing with the different type of data being integrated and thus annotated in the analysis. In particular, we can observe that the joint analysis (Table 2, columns 2-4) gives fully relevant molecular information only when all 3 types of molecules are being annotated. In fact, although pigmentation during development, *pigmentation*, *melanocyte differentiation*, *pigment cell differentiation* and *melanin metabolic process* are constantly statistically significant, *Melanogenesis* and *melanin metabolic process* only appear when proteins or proteins and miRNAs are included for annotation. Compared to the separate analysis -although both methods give complete molecular information on biological processes related to *pigmentation during development*, *pigmentation*, *melanocyte differeniation* and *pigment cell differentiation*- the joint analysis enriches the description of *Melanogenesis*, that is the major process upstream the *melanocyte differentiation* and *pigmentation*. Conversely, the separate analysis cannot report as enriched the *melanin biosynthetic process* and *melanin metabolic process*, which are processes related to the basal melanocyte physiology (see Table 2, columns 5-7).

**Table 2:** Comparison of the functional annotation results from different methods for Melanoma

		**Molecules**						
		**Joint Analysis FA (HC)**				**Separate Analysis FA (HC)**		
		**mRNA**	**mRNA**	**mRNA**		**mRNA**	**mRNA**	**mRNA**
			**&Prot**	**&Prot**			**+Prot**	**+Prot**
**Biological terms**				**&miRNA**				**+miRNA**
GO:0048066 pigmentation during development		X (*X*)	X (*X*)	X (*X*)		X (*X*)	X (*X*)	X (*X*)
GO:0043473 pigmentation		X (*X*)	X (*X*)	X (*X*)		X (*X*)	X (*X*)	X (*X*)
GO:0030318 melanocyte differentiation		X (*X*)	X (*X*)	X (*X*)		X (*X*)	X (*X*)	X (*X*)
GO:0050931 pigment cell differentiation		X (*X*)	X (*X*)	X (*X*)		X (*X*)	X (*X*)	X (*X*)
GO:0042438 melanin biosynthetic process		X (*X*)	X (*X*)	X (*X*)		- (*X*)	- (-)	- (-)
GO:0006582 melanin metabolic process		- (-)	X (*X*)	X (-)		- (-)	- (*-*)	- (-)
GO:0046148 pigment biosynthetic process		- (-)	- (*X*)	- (-)		- (-)	- (-)	- (-)
hsa04916:Melanogenesis		- (*-*)	X (-)	X (-)		- (-)	- (-)	- (-)
BP00193:Developmental processes		- (-)	X (-)	- (-)		- (-)	X (*X*)	- (-)
GO:0010033 response to organic substance		- (-)	X (-)	- (-)		- (-)	- (-)	- (-)
GO:0019233 sensory perception of pain		- (-)	X (-)	- (-)		- (-)	- (-)	- (-)
GO:0030029 actin filament-based process		- (*X*)	- (*X*)	- (-)		- (*-*)	- (-)	- (-)
GO:0030036 actin cytoskeleton organization		- (*X*)	- (*X*)	- (-)		- (-)	- (-)	- (-)
GO:0001501 skeletal system development		- (-)	- (-)	- (-)		- (-)	X (*X*)	- (-)

Comparison of the annotations done on the joint versus the separate analysis for the FA based method (indicated with FA). The table also contains the comparison with the alternative method hierarchical clustering and SAM (indicated by *HC*, the results are listed in parentheses in each cell and refer to the application of HC to the joint or separate analysis coherently with the FA annotation in the same cell). &indicate that the annotations are done on the corresponding molecules treated jointly and + indicates that annotation is done downstream of 3 independent analyses.

### 

#### 

##### Relevance of miRNAs in Melanoma

The miRNA list identified by the proposed integrated method are shown in the worksheet **Joint** in Additional file 1. Our results indicate that miR-204 and miR-211 are important in melanoma cell lines and this is consistent with specific tumor profiles previously reported [23]. In particular, miR-211 transcription is described to be regulated by the microphthalmia-associated transcription factor (MITF), a master switch of melanocytes development and melanoma progression via Wnt/*β*-catenin signaling. In a deeper investigation [37] an additional mechanism of action is proposed: MITF transcriptionally induces miR-211 to inhibit the translation of POU3F2/BRN2 (POU class 3 homebox 2), therefore increasing the invasive potential of tumor cells. Consistently, in our analysis MITF as well as POU3F2 appear to be relevant in the melanoma. Sakurai and colleagues [38] found that miR-211 participates to the expression of Preferentially Expressed Antigen of Melanoma (PRAME, c23). In our case, PRAME is identified as key gene and the functional analysis results show that it may work in the apoptosis/cell death and proliferation processes. Moreover, in our results cell death and apoptosis emerge as related to the presence of miR-363 and miR-146a. High levels of miR-146a were in fact revealed in the melanoma cell lines, and their function is known to be related to their metastatic potential [23]. Finally, we identified a set of miRNAs in the miR-509-miR-514 cluster, including miR-509-3-5p, miR-509-3p, miR-509-5p, miR-513c and miR-514. Comparing to other tissues, all these miRNAs showed a high level of expression in melanomas, consistent with literature findings [23,39]. This miRNA cluster is located on Xq27.3 in the human genome, very close to the Melanoma Antigen family A genes (MAGEA1, MAGEA4 and MAGEA8) and CSAG2 (CSAG family, member 2), which are key mRNAs and expressed at a high level in our data. Therefore, this miRNA cluster, along with melanoma associated antigens, is likely to be cis-transcribed and may represent a molecular signature able to distinguish melanoma from all other tumor tissues. The separate analysis highlighted two more miRNAs: miR-224 and miR-502-3p, which are melanoma-relevant. However, no connections between mRNAs/proteins and miRNAs were found.

##### Emergence of the Wnt Pathway in Melanoma

We then turned our attention to the genes known in literature to be related to melanoma. In particular, human pigmentation appears to be one of the main modulators of individuals’ risk of developing malignant melanoma [40]. Among the relevant genes we identified, Dopachrome Tautomerase (DCT) is reported to play a critical role in lowering the oxidative stress melanocytes are physiologically subjected to during pigmentation; it is also known that levels of DCT are elevated in melanoma cell lines which are particularly resistant to chemotherapy and radiation [41]. Edn Receptor Type B (EDNRB) is another relevant gene essential for the development of melanocytes and has been associated with melanoma progression [42]. Finally, Tyrosinase (TYR) and Tyrosinase Related Protein 1 (TYRP1/gp75), two proteins involved in the melanocyte pigmentary machinery, are increasingly used as differentiation markers given their emerging role in malignant transformation and tumor progression [43]. In our results, these genes all contribute to the emergence of the *Melanogenesis* annotation, the physiological process driving differentiation of neural crest progenitors, their migration and maturation into functional melanocytes. Consequently, we chose to investigate the connection among all the genes related to this annotation, making use of STRING (http://string.embl.de/, [44]). This database of known and predicted protein interactions, includes direct (physical) and indirect (functional) associations derived from four sources: genomic context, high-throughput experiments, coexpression and literature. The 4 sources can be further extended into 8 types of evidences and in a STRING map, edges’ color represent the different types of evidence. The confidence score (set here to the default value *medium*) is an indicator of the robustness of the connection.

**Figure 2:**
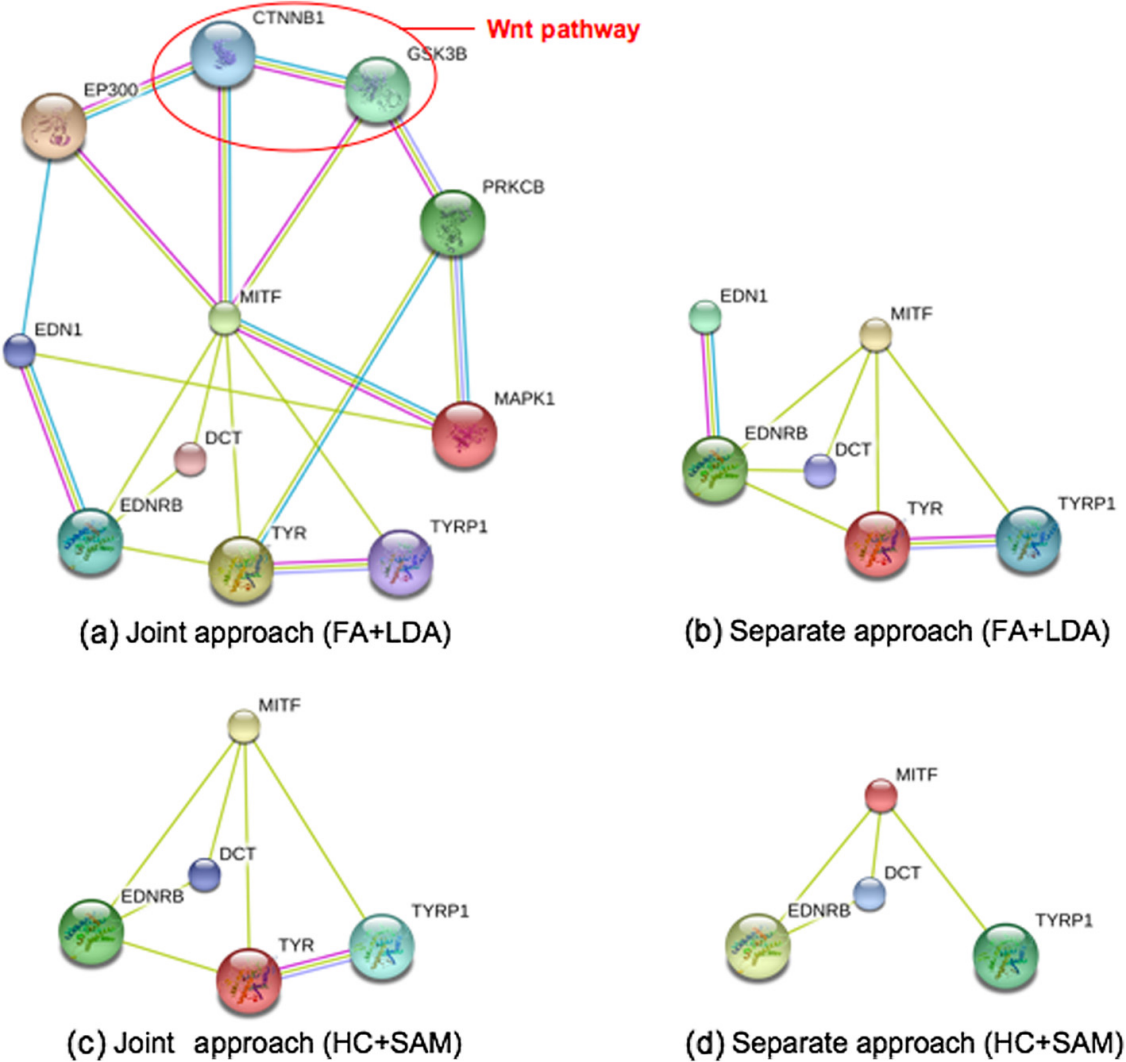
**Melanogenesis.** Network of interaction among the molecules related to Melanogenes using STRING (http://string.embl.de/) obtained from different methods, including joint and separate analysis based on FA+LDA (combination of factor analysis and linear discriminant analysis) and HC+SAM (combination of hierarchical clustering and SAM). **(a) Joint approach (FA+LDA)** and **(b)Separate approach (FA+LDA)** are from the joint and separate analyses based on FA+LDA methods, respectively. The loss of connectivity due to the lack of molecules CTNNB1 and GSK3B in the separate analysis corresponds to a loss of information related to the Wnt signaling pathway which is of utmost relevance in melanocyte differentiation and melanoma onset. **(c) Joint approach (HC+SAM)** and **(d) Separate approach (HC+SAM)** illustrate the results from the joint and separate analyses based on HC+SAM methods, respectively. Similarly to the FA+LDA methods, joint analysis shows better performance than the separate analysis since the latter is not able to identify the key factor *TYR* for melanogenesis. Overall, regarding melanogenesis, FA+LDA methods outperforms HC+SAM, and the joint analysis is more informative than the separate analysis. Joint analysis based on FA+LDA only is able to uncover the *emergent* melanogenesis process in melanoma.

As it is shown in Figure 2, the mild increase in the number of molecules between the joint and separate analysis (11 in Figure 2(a) versus 6 in Figure 2(b)) is nonetheless able to drastically change the informative content of the findings. Genes CTNNB1 and GSK3B emerging in the joint analysis are of particular relevance. These tightly interacting molecules related to the Wnt canonical pathway, are known to crucially regulate melanoblasts fate [45] and even to be involved in melanoma [46]. CTNNB1 and GSK3B genes codify for protein *β*-catenin and its repressor, Glycogen Synthase Kinase 3-*β*, respectively. Notably the former is the key factor of the highly conserved canonical Wnt signaling pathway, which activation culminates in the *β*-catenin cytosolic accumulation and nuclear translocation. Then its interaction with transcription factors results in the regulation of target genes mediating cell fate, proliferation, and migration. Mutations or aberrant expression of canonical Wnt pathway components, have been identified to promote deregulation of *β*-catenin-responsive genes affecting cell differentiation and apoptosis, and are thus responsible of tumor initiation and progression. In particular in colon and liver cancers canonical Wnt signaling produces enhanced quantities of cytoplasmic and nuclear localized *β*-catenin, which correlates with invasion and poor prognosis. Conversely *β*-catenin in melanoma is associated with good outcome and improved survival, while its reduced expression is linked to cancer progression, including metastasis. A wide range of studies have validated the immunohistochemical detection of nuclear *β*-catenin as a survival marker in several cancers, solidifying the importance of this pathway in oncogenesis and in tumor progression [47]. Since increased nuclear *β*-catenin is found in the majority of benign nevi and in tumors with low proliferative index, it has been considered as a surrogate marker of cell differentiation and useful to identify the histological phenotype of tissue lesions [48,49].

What is unique to the joint analysis (see Figure 2(a)), is that, within Melanogenesis the contribution of the Wnt/*β*-catenin signaling pathway emerges. Without gene CTNNB1 (missing in the separate analysis, Figure 2(b)) it is not possible to mention the canonical Wnt signaling pathway and therefore, all the above considerations, that are crucially related to the characterization of melanoma and carcinogenesis, have to be ignored. In summary through our computational results we can conclude that the joint approach is able to obtain more information then the separate one, from the same data. As a consequence, our findings can be informative on the mechanism underlying the biology of tumors and therefore contribute to understanding the nature of the neoplastic lesion, which is of crucial importance to identify a suitable and effective therapy.

### Comparison with other integrated approaches

Preprocessing, definition of joint and separate analyses and the method used to evaluate the significance of the discrimination (*χ*^2^-test) are the same used in the FA based-method. Results on the annotations are listed in parentheses in Table 2.

#### 

##### Alternative Joint Analysis

The hierarchical clustering results show that the joint analysis can significantly discriminate the tissues with global accuracy 0*.*62 (p-value <10^−7^) lower than the FA based method (0*.*83). The accuracy and p-values for discriminating individual tissues from others are CNS (1,2×10^−12^), CO (0*.*93,1×10^−11^), LE (0*.*95,9*.*4×10^−14^), ME(0*.*98,1×10^−11^), LC(0*.*93,1×10^−7^), RE(0*.*98,4×10^−11^). For a detailed and fair comparison SAM was used to select the most differentially expressed molecules, through comparison of the melanoma cluster with all other tissues. SAM identified an heterogeneous signature of 159 mRNAs, 2 proteins and 21 miRNAs for melanoma (*FDR*≤0*.*001, *Δ*=2*.*6). Similarly to the FA-based joint analysis, mRNAs and proteins were significantly enriched in the biological terms *pigmentation during development*, *pigmentation*, *melanocyte differentiation*, *pigment cell differentiation* and *melanin biosynthetic process*. However, the important pathway *melanogenesis* did not appear to be significantly enriched. Only five genes, DCT, EDNRB, MITF, TYR and TYRP1 are found in the melanogenesis pathway, again missing the essential Wnt signaling pathway genes: CTNNB1 and GSK3B (see Figure 2(c)). Comparing to the FA joint analysis, more miRNAs (21 versus 14) were identified. In particular, the miR-509-514 cluster is shared with the FA joint analysis, but no nearby genes, such as MAGEA1, MAGEA4, MAGEA8 and CSAG2 were identified in the list of key mRNAs, nor proteins.

##### Separate Analysis

Using hierarchical clustering, both mRNAs and miRNAs can perfectly discriminate ME from other tissues (0*.*98,1×10^−11^). Conversely, proteins alone are not able to identify ME and therefore we did not apply SAM to this dataset. On the contrary, on mRNAs, SAM was able to identify 149 molecules, significantly enriched in *pigmentation during development*, *pigmentation*, *melanocyte differentiation* and *pigment cell differentiation*. As in the clustering joint analysis, the melanogenesis pathway information did not emerge as an enriched one, and only DCT, EDNRB, MITF and TYRP1 were included, see Figure 2(d). Regarding the miRNAs, 20 molecules -most of which are shared by the two (joint and separate) analyses- are found to characterize ME, meaning that no additional nor diminished information appears when comparing miRNA results to the joint analysis. Considering the nearby genes of the miR-509-miR-514, only MAGEA6 (Melanoma Antigen family A 6) and LOC100130935 (CSAG2) located at Xq28, which are also highly expressed in ME, are found in the mRNA list.

## Application of the TCGA dataset

To asses our approach not only in terms of the improved knowledge obtained from the joint versus separate analysis, but also in terms of the relevance of the information carried by the latent features, we applied the method to a more complex dataset that is a large high-grade serous ovarian adenocarcinomas dataset (HGS-OvCa)[4]. For each patient several clinical parameters are provided, namely: *AgeAtDiagnosis*, *VITAL STATUS*, *TUMORSTAGE *, *TUMOR GRADE*, *Platinum Status*, *TUMORRESIDUAL*, *PRIMARYTHERAPYOUTCOMESUCCESS*, *OverallSurvival*, *ProgressionFreeStatus* and *ProgressionFreeSurvival*. The dataset is extremely rich and complex, including also methylation and copy number variation data (but no proteins). In order to perform a fair validation of the above method we only used mRNA and miRNA data, which could nevertheless recollect important clinical information found in the original publication. In the near future we plan to include other omic layers (methylation/copy number) upon evaluation of the impact of the different data distribution (binomial and discrete respectively).

We downloaded 489 mRNA and miRNA profiles from HGS-OvCa patients. Among these 489 patients, we retained only the 287 that have defined information of the response to platinum treatment (PS, *PLATINUM STATUS*). The mRNA dataset is obtained from 3 platforms: Affymetrix Exon 1.0, Agilent 244k Whole Genome Expression Array and Affymetrix HT-HG-U133A, as described in Supplementary Methods S6 of [4]. Gene expression values were rescaled as relative gene expression values, calculated by subtracting the mean expression value across samples from the gene estimate and dividing by the standard deviation across patients. To join the mRNA and miRNA dataset, we calculated the relative gene expression value for miRNAs in the same way as mRNAs. The FA+LDA approach let emerge a 13-factor model (M 13) which correlates with important aspects of the clinical outcomes i.e. *PLATINUM STATUS*, that is the response to the platinum-based chemotherapy from the date of last primary treatment, and *VITAL STATUS*, or the living/deceased patients status at follow-up. In particular, among the 13 factors in M 13, F 7 can discriminate platinum resistance from platinum sensitivity with accuracy 0.7 and F 8 can discriminate both Living and Deceased patients significantly from all other patients with accuracy 0.635 and 0.632, respectively.

DAVID functional annotation of the genes identified within M 13 revealed several significantly represented biological categories related to HSG ovarian cancer (HSG Ov-Ca), see Additional file 2 for the details of the key molecules and the enriched biological terms. From a general point of view functions like *Immunity*, *Antigen presentation* and *Inflammatory* response are known to be strictly connected and to play a fundamental role in the antitumoral immune activity [50,51]. Similarly, physiological processes like *Development*, *ECM-interaction* and *Plasminogen cascade*, normally regulating tissue remodeling, lead to cancer growth and spreading through metastases, when altered [52]. We found F 7 and F 8 of particular relevance, as they are able to describe essential and peculiar aspects of HSG Ov-Ca and they correlate with clinical indexes referring to chemotherapy efficacy such as resistance/sensitivity to platinum treatment and patients survival respectively. Specifically, most of the enriched pathways characterizing F 7 are related to *Development and Morphogenesis*. All the embryonic developmental processes such as *Ectoderm development*, *Neurogenesis*, *Developmental processes*, *Embryonic skeletal system development and morphogenesis*, *Anterior/posterior pattern formation* share biological terms belonging to the HOX family of homeobox genes. The precise spatial and temporal expression of these genes is well acknowledged to be critical in specifying organ patterning of the reproductive tract during embryogenesis, and in controlling proliferation, cell migration and DNA repair. Aberrant activation of such embryonic pathways is implied in the neoplastic transformation of ovarian cancer tumorigenesis [53]. Several studies describe the HOX genes family as able to influence HSG Ov-Ca subtypes development, their aggressiveness and the likelihood of metastasis together with the response to therapy, as such they are biomarkers investigated in histopathology [54,55]. In addition to the HOX gene network an important transcription factor of embryonic patterning, RUNX3(runt-related transcription factor 3), was found to be differentially expressed within F 7. RUNX3 has been reported to be overexpressed in HSG Ov-Ca cells and tissues, upregulating cells proliferation through downstream interference with TGF-*β*(transforming growth factor beta) cellular growth inhibition [56]. It is noteworthy that RUNX3 immuno-staining in HSG Ov-Ca subtypes samples correlate with clinical-pathological variables, like overall survival of platinum treated patients [57]. Hence RUNX3 is a key molecule acting as prognostic factor for HSG Ov-Ca characterization, since is involved in platinum resistance mechanisms.

Among the miRNAs let-7b and miR-203 in F 7 are noteworthy (see sheet **miRNA lists** in Additional file 2). In fact, let-7b and miR-200 families are well acknowledged as two major microRNA families frequently deregulated in ovarian cancer and associated with tumor aggressiveness, tumor invasion and chemoresistance [58,59].

**Figure 3:**
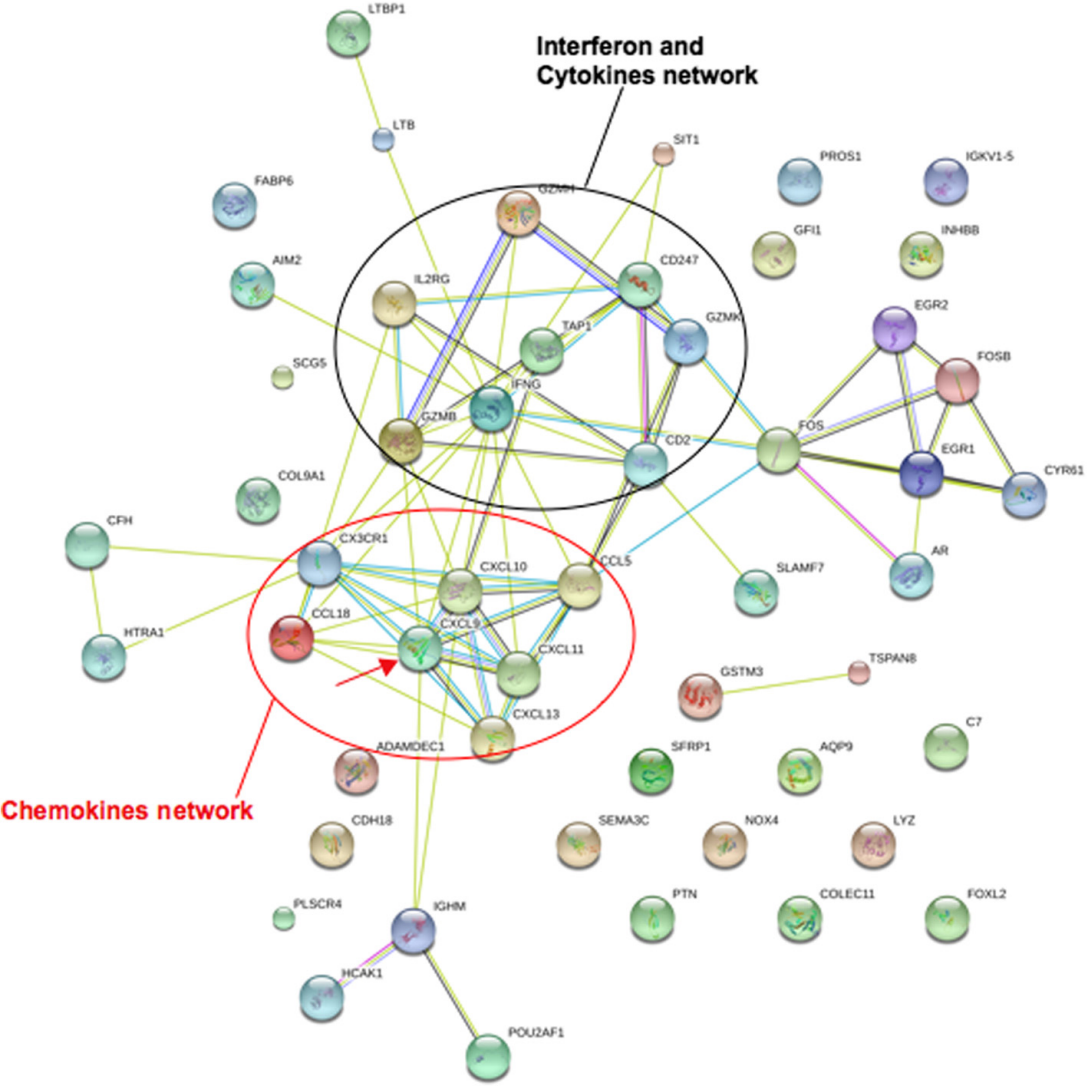
**Interactions of genes relevant to the clinical response to Platinum treatment in TCGA.** Interactions of the relevant genes in F 8 are reconstructed using STRING (http://string.embl.de/). Highlighted are the chemokines family (red oval) and the Interferon and cytokines (black oval) networks. Of remarkable importance is the central role played by CXCL9 (red arrow) in orchestrating the immune and inflammatory responses, which correlate with the platinum therapy efficacy.

The other relevant factor, F 8 was found to be enriched for biological processes/pathways such as *Immune response*, *Cytokine/chemokine (eg. ILs, CXCLs), Interferon (IFNs) and Macrophage mediated Immunity*, *Antigen presentation* and *Inflammation*, based on the functional analysis on both mRNAs alone and mRNAs and miRNAs jointly (see sheet **FuncAnnos of mRNA&miRNA**, Additional file 2). Network representation from STRING [44] of the genes involved in these biological processes/pathways show that the relevant genes highlight the chemokines family (red oval in Figure 3) and Interferon and cytokines (black oval in Figure 3). These findings are of high relevance to HSG Ov-Ca, since immunity and inflammatory cytokines stimulation have been clearly proven to mainly influence either the tumor phenotype or the platinum chemotherapy response [60]. Moreover, in an elegant large-scale study Yoshihara and colleagues [61] compared with different approaches two sets of data with the TCGA dataset here analyzed and found the same set of overrepresented pathways [4]. They established a HSG Ov-Ca gene signature consistent with the TCGA study results, and also found a significant correlation between this signature and the platinum treated patients overall survival. Most of the immune related signaling pathways these genes belong to, emerge in our results as well. In particular CXCL9(chemokine (C-X-C motif) ligand 9) (highlighted by a red arrow in Figure 3) is overrepresented in all the biological processes enriched in F 8. Interestingly, this gene belongs to the molecular signature they defined as predictive of platinum therapy response. Additionally they demonstrated that alterations to the immune system in cancer cells are one of the most important factors affecting survival of patients with HSG Ov-Ca and that, in particular, high-risk ovarian cancers are well characterized by alterations of the immune activity such as downregulation of the antigen presentation pathway. In fact, defects in the HLA antigen presentation machinery are known to decrease recruitment of tumor-infiltrating lymphocytes, leading to poor prognosis in cancer patients because of a reduction in antitumor immune activity [62]. Also, inflammation mediated immunity, like Interferon or other cytokines stimulation, plays a central role in response to the therapy since it regulates the expression of genes in the antigen presentation signaling [63].

Concerning the miRNA list identified by F 8 (see sheet **miRNA lists** in Additional file 2) we found miR-30d*, miR-30b*, miR-155 and let-7f-2* most related to HSG Ov-Ca. miR-30d* is of particular relevance since it has been significantly associated with clinico-pathological indexes, as platinum treated patients’ disease-free or overall survival [64]. Among the others, miR-155 is known to be differentially expressed in the ovarian cancer tissue and serum samples [65], whereas miR-30b* and let-7f-2* are reported to regulate ovarian cancer cells proliferation and viability [66,67].

## Conclusions

We have shown how the use of integrated data and further processing with FA can enhance the power of the analysis and give more insight than separate approaches, based on the same original information. In particular, future work is warranted for the integration of additional *omic* levels, among which the genomic level, for example replacing our approach to the correlation used in [25] to identify mutations drivers in cancers, and importantly for the integration of epigenomic data, which binomial distribution strongly differs from expression data.

## Competing interests

The authors declare that they have no competing interests.

## Authors contributions

YL analyzed the data. SC and VD interpreted the cancer characteristics. YL, VD and CN wrote the manuscript. CN designed and coordinated the study and revised the manuscript. All authors read and approved the final manuscript.

## Supplementary Material

Additional file 1 **Key molecules of melanoma identified using the FA based joint and separate analysis for NCI datasets.** This.xls file has two sheets.The first one, named **Joint**, listed the molecules identified using the FA based integrated method (joint analysis). All the molecules are identified as a whole, but divided into three groups with the headers mRNA, miRNA and protein, respectively, in this sheet. The second sheet, named **Separate**, includes the molecules identified using the separate method. Each column represents one set of molecules resulted from one single omic dataset. Click here for file

Additional file 2 **Key molecules and functional annotations for the factors in M 13 resulted from TCGA datasets.** This.xls file has four sheets. The first one, named **mRNA lists**, listed the mRNAs identified for the factors (from F 1 to F 13) in M 13 using the FA based integrated method (joint analysis), each column includes the key mRNAs for one factor. The second sheet, named **miRNA lists** is to list the miRNAs for each factor. The third sheet, named **FuncAnnos of mRNAs**, are the functional analysis results of mRNAs, where the mRNAs of each factor are annotated using DAVID annotation tool to identify the significantly enriched terms. The last sheet, named **FuncAnnos of mRNAs&miRNAs**, are the functional annotations of the integration of mRNAs and miRNAs for F 7 and F 8, where mRNAs and miRNA targets predicted using *TargetScan* are merged for DAVID functional analysis. Click here for file
